# Relationship of Maxillary Sinus Volume and Nasal Septum Deviation: A Cone Beam Computed Tomography Study

**DOI:** 10.3390/diagnostics14060647

**Published:** 2024-03-19

**Authors:** Amanda B. Rodriguez Betancourt, Leidy J. Martinez Somoza, Carlos Romero Mesa, Tolga Fikret Tozum, Carlos Fernando Mourão, Jamil Awad Shibli, Lina J. Suárez

**Affiliations:** 1 Departamento del Sistema Periodontal, Facultad de Odontología, Pontificia Universidad Javeriana, Bogotá 110231, Colombia; arodr368@uic.edu (A.B.R.B.); martinez.lj@jaaveriana.edu.co (L.J.M.S.); lsuarez@javeriana.edu.co (L.J.S.); 2 Department of Periodontics, College of Dentistry, University of Illinois Chicago, Chicago, IL 60612, USA; ttozum@uic.edu; 3 Instituto Roosevelt, Hospital Universitario, Bogotá 11023, Colombia; cromero_mesa@hotmail.com; 4 Department of Periodontology, Tufts University School of Dental Medicine, Boston, MA 02111, USA; 5 Dental Research Division, Department of Periodontology, Guarulhos University, Guarulhos 07001, Brazil; jshibli@ung.br

**Keywords:** maxillary sinus volume, septal deviation, computed tomography

## Abstract

The present study was designed to test the hypothesis that there would be a correlation between nasal septum deviation (NSD) and a decreased maxillary sinus volume (MSV) in a Colombian population, using Cone Beam Computed Tomography (CBCT); other sinusal anatomical structures found during the reading were described and analyzed. A retrospective analysis of 537 CBCT scans of adult patients taken between January 2014 and January 2017 included measuring the maxillary sinus diameter in the vertical, horizontal, and sagittal planes. NSD was quantified and related to MSV using the same field of view (FOV). The volume of the right and left maxillary sinuses showed a median and interquartile range (IQR) of 8.18 mm^3^ (IQR: 6.2–10.33) and 8.3 mm^3^ (IQR: 6.4–10.36). Statistically significant differences were observed between sex and right and left MSV (*p* = 0.000), with higher MSV in men. The presence of NSD was observed in 96.81% of the sample and was evaluated in degrees, observing a median of 11° (IQR: 7–16) where 40% of the sample had moderate angles (9–15°). There was no correlation between NSD and a decreased MSV in the population studied. Detailed CBCT analysis with a large FOV is crucial for the analysis of anatomical structures before performing surgical procedures that involve the MS as a preventive diagnostic and therapeutic step for appropriate treatment.

## 1. Introduction

The maxillary sinus (MS) is the first of the paranasal sinuses [1]. It develops in the tenth week of fetal life through a mucous sac and is formed mainly by the invagination of the mucosa of the middle nasal meatus. Its shape varies from spherical to an irregular pyramid-shape, covered by stratified cylindrical mucosa and drained through the ostium. The MS morphology analysis must include the volume and degree of lateral pneumatization to the zygoma. Its size and volume depend on the development of surrounding structures and mechanisms such as nasal airflow, brain growth, muscle mass traction resulting from the action of the facial muscles, facial structures, and cellular mechanisms of adherence and migration [1,2,3,4,5,6,7]. Due to its location within the midface skeleton, measuring its volume is a complex procedure in general clinical practice [4,5]. As a diagnostic method, Cone Beam Computed Tomography (CBCT) is one of the most frequently used methods for characterizing the MS; however, there are factors that hinder the precision and reproducibility of the measurement due to the location of the internal margin of the paranasal cavity. The mean volume can be highly variable and ranges between 8.6 and 24.9 cm^3^ [2,3,6,8].

The nasal septum (NS) constitutes the medial wall of the nostrils and favors laminar flow. It is made up of bone and cartilaginous components. Septal deviations can originate during development or by trauma and could affect the maxillary sinus volume (MSV), considering its relationship with surrounding anatomical structures such as the hard palate and lateral wall of the nasal cavity [9]. Nasal septum deviation (NSD) may potentially contribute to sinus disease by narrowing the osteomeatal complex which could affect the amount of adequate ventilation [10,11,12]. NSD can cause nasal obstruction by increasing nasal airway resistance and causing turbulent nasal airflow, which precipitates pathologic conditions such as dryness and crusting of the nose, frequent nosebleeds, and leads to recurrent sinusitis by impaired mucociliary clearance [11]. Simultaneously, the impaired nasal breathing of NSD patients can lead to chronic mouth breathing, resulting in maxillary constriction, and other maxillofacial and temporomandibular changes [13]. The relation between NSD with MSV have been evaluated in different populations, and a direct relationship has been found; there are results supporting the findings that NSD led to a reduction in MSV towards the side of the deviation [1,6,14,15,16,17,18], but the evidence on how the nasal septum could act as a potential contributor to these changes in MSV is contradictory [5,6,14,15,17].

As a first stage of planning an MS floor elevation procedure, it is mandatory to make a preventive diagnosis of the different structures and conditions, with the purpose of establishing a pre-therapeutic ear–nose–throat (ENT) treatment that will avoid sinus lift-related sinonasal complications. “Potentially reversible ear, nose, and throat contraindications” including Anatomic-structural alterations must be identified and evaluated [19,20,21,22]. The factors to be identified include those that potentially change the drainage-ventilation pathways in the maxillary sinus among them, septal deviation [19,21]. Hence, emphasis should be placed on the CBCT diagnosis and multidisciplinary treatment of patients with craniofacial anomalies (including nasal septum deviations) that could affect sinus volume and could be a predictor of future surgical complications [3,23]. The aim of this study was to measure the volume of the maxillary sinus in a Colombian population, by analyzing computed tomography scans using a mathematical model and estimating the prevalence of nasal septum deviations and their relationship with the decrease in maxillary sinus volume.

**Hypothesis 1 (H1).**  *There would be a correlation between nasal septum deviation and a decreased maxillary sinus volume.*

**Hypothesis 2 (H2).**  *There would be no correlation between nasal septum deviation and a decreased maxillary sinus volume.*

## 2. Materials and Methods

### 2.1. Sample Population

The data collection for this retrospective descriptive observational study was conducted in accordance with the Declaration of Helsinki, and the protocol was approved by the Research and Ethics Committee of the Dental Faculty of the Pontificia Universidad Javeriana (Bogota, Colombia, OD-0281). A total of 537 CBCT scans performed over the course of 4 years (from January 2014–January 2017) were provided by the Salitre radiological center in Bogotá, Colombia. In all cases an ACCUITOMO tomograph model 170 was used to take the images, with 80 kV and 5.0 mA. exposure. The minimal field of view was fixed at 140 mm in diameter and 100 mm in height which is a wide window size that allowed observation of all the anatomical structures necessary for measurement of the structures evaluated in the study. The inclusion criteria were CT scans of subjects older than 18 years old, without distortion and in good condition, in which the structures to be evaluated were clearly observed, i.e., nasal septum and maxillary sinuses. Subjects with a history of functional endoscopic sinus surgery were not excluded. Subjects were excluded if they presented pathologies that altered the walls of the maxillary sinuses.

### 2.2. CBCT Image Analysis

All CBCT scans were assessed with the use of specific software (One Volume Viewer Software, i-Dixel 3DX Vision 2.2.1.3T, J. Morita, Kyoto, Japan). The images were reproduced and observed in the axial, coronal, and sagittal planes. All measurements were made to determine the maxillary sinus volume and deviation of the nasal septum and to detect abnormalities in the paranasal complex. To determine the reproducibility and reliability of the variables measured, an intra-examiner and inter-examiner calibration was performed and an intraclass correlation coefficient (ICC) was used to compare the examiner in relation to reference standards with a strength of agreement >0.75. All measurements were taken by 2 experienced and calibrated professionals (A.B.R. and L.J.M.) and any discrepancy was resolved by an E.N.T specialist (C.A.R.).

### 2.3. Image Analysis

**Figure 1:**
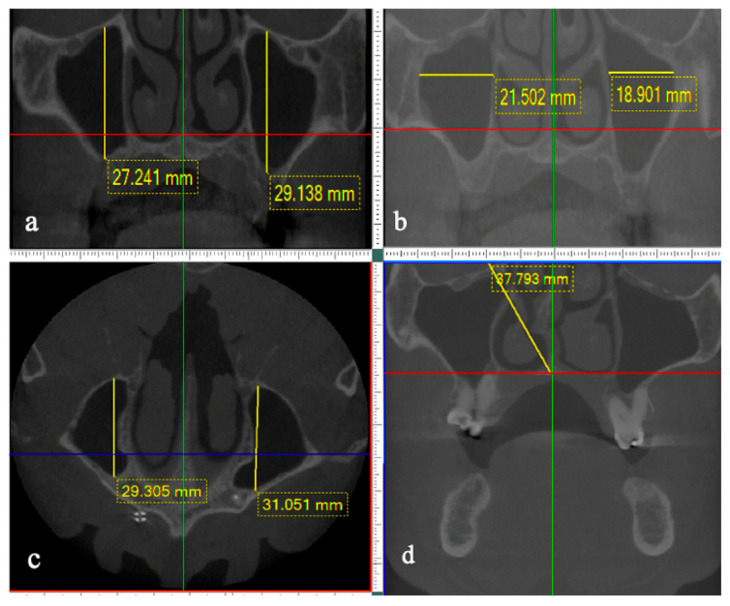
Measurement of the maxillary sinus: height (**a**), width (**b**), depth (**c**), and the angle of deviation of the nasal septum (**d**).

Image measurements (in mm) were performed manually on CBCT scan images as previously described [5]. Briefly, diameter in the vertical plane, width in the horizontal plane, and length in the sagittal plane were evaluated. MS height in the vertical plane (maximum craniocaudal diameter) was defined as the longest distance from the lowest point of the lower wall to the highest point of the upper wall (Figure 1a), width in the horizontal plane (maximum transverse diameter) was defined as the longest perpendicular distance from the most prominent point of the medial wall to the most prominent point of the lateral wall (Figure 1b) and length of the MS in the sagittal plane (maximum anteroposterior diameter) defined as the longest distance from the most anterior point of the anterior wall to the most posterior point of the posterior wall (Figure 1c). Subsequently, the shape of the MS was established, similar to the forms proposed [5], irregular pyramid or sphere. Manual anatomy and radiology data were analyzed. This morphology was classified by an expert and trained E.N.T Doctor (CR). Calculations were made for the volume called “Manually calculated maxillary sinus volume,” considering the previously established shape for each MS and according to the following formulas:

1. V = 4/3 r3. It was called the mMSV “sphere”.
2. V = 1/3 A × h. It was called the mMSV “pyramid”.

NS deviation was defined as any curvature in the contour of the nasal septum observed in the coronal CBCT images. To quantify the septal deviation, the measurements of the maxillary sinus volumes and the septal deviation angles were standardized using the same field of view (FOV) [13,14,15,23]. The angle was determined, taking as a reference the anterior nasal spine projecting towards the upper cranial third to establish a baseline together with another line that started from the anterior nasal spine to the outermost edge of the septal deviation (Figure 1d) [15]. In patients with NSD, the MSV was compared on the deviation side with the non-deviation side on the same subject, subgroup comparisons were made according to the magnitude of the deviation. The presence of pathologies and findings of the MS were reviewed in the coronal, sagittal, and multiplanar axial slices, and the findings were recorded as retention cysts, mucoceles, membrane thickening, ostium blockage, increased ostium, paranasal obstruction, maxillary hypoplasia, hypertrophy of the inferior turbinate, presence of graft material, partial, unilateral or bilateral, random or total edentulism, presence of septa. Total and partial edentulous spaces were recorded on axial, coronal, and sagittal images, and the volumes of the MS were compared on the side of the missing tooth and the contralateral side.

### 2.4. Statistical Analysis

Shapiro–Wilk and Kolmogorov–Smirnov tests were performed to evaluate normality (*p* < 0.005), since a normal distribution of the variables was not observed for the variables volume of the maxillary sinus and angulation of the nasal septum. Mann–Whitney U and Kruskal–Wallis tests were performed for the bivariate analysis of quantitative variables. When dividing the sizes of the volumes into three groups, Chi-square and Fisher were used with respect to the other variables. A < 0.05 was considered significant with a significance level of 95%.

## 3. Results

Out of the sample of 537 bilateral maxillary sinus and nasal septum CBCTs, 470 scans met the selection criteria, of which 177 (37.66%) correspond to male subjects and 293 (62.34%) to female subjects. The remaining scans were excluded due to the absence of clear limits to obtain measurements for MSV calculation.

### 3.1. Maxillary Sinus Volume Analysis

Height, width, and depth measurements were taken of each of the sinuses evaluated to determine the volume by using a mathematical model. For these non-parametric measurements, a median of 30.48 mm (interquartile range (IQR): 27.38–33.84) was observed for the right sinus, 24 mm wide (IQ: 20.82–26.76) and 33.6 mm depth (IQ: 30.3–36.16) and for the left sinus a height of 30.74 mm (IQR: 27.65–34.11), width of 24.32 mm (IQR: 21.59–26.94) and depth of 33.52 mm (IQR: 30.56–36.35). The shape of all the sinuses was found to be pyramidal. The volume of the maxillary sinuses for the right and left sides showed a median and IQR of 8.18 mm^3^ (IQR: 6.2–10.33) and 8.3 mm^3^ (IQR: 6.4–10.36), respectively. MSV was classified into tertiles: low (<6.95 mm), medium (6.96–9.50 mm), and high (>9.50 mm). When comparing the tertiles of the right and left sinus volumes, values of 0.95 to 6.95 mm were observed for the low tertile on the right side; for the middle tertile from 6.97 to 9.49 mm, and for the high tertile from 9.54 to 16.58 mm. For the left side, the values were from 0.51 to 6.94 mm in the low tertile, medium from 6.97 to 9.51 mm, and high from 9.54 to 18.77 mm. The results of the Spearman test showed a positive and strong correlation between the volume of the right sinus and the volume of the left sinus (Rho = 0.8; *p* = 0.001) with no statistically significant differences. However, the volume of the left maxillary sinus showed an increased value when compared with the right MS (Table 1).

### 3.2. Relationship between Sex and Left and Right Maxillary Sinus Volume

**Figure 2:**
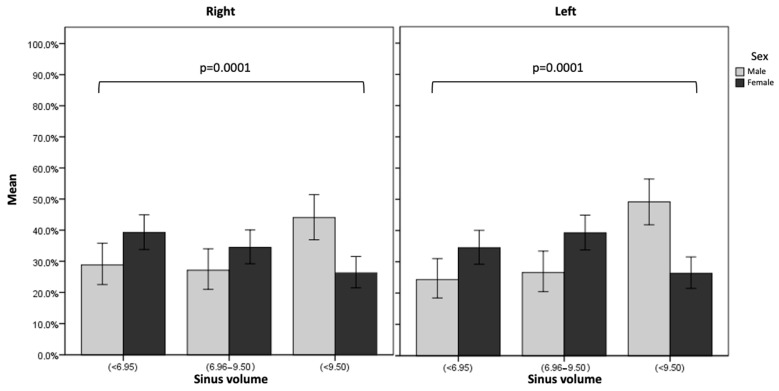
Relationship between left and right maxillary sinus volume tertiles with sex.

Statistically significant differences were observed between sex and right and left MSV (*p* = 0.000), with higher maxillary sinus volume in men compared to women. In women, MSV for the right and left sides showed a median and interquartile range (IQR) of 7.7 mm (IQR: 5.8–9.6) and 7.9 mm (IQR: 6.1–9.7), respectively, whereas for men, MSV for the right and left sides showed a median and interquartile range (IQR) of 9 mm (IQR: 6.8–11.7) and 79.4 mm (IQR: 7.2–11.4), respectively. Additionally, statically significant differences between sex and left sinus volume tertiles (*p* < 0.05) were observed. In women, a higher proportion of patients were observed in the low category, shown in 101 sinuses (34.47%), and in the middle category in 115 sinuses (39.25%). In men, the highest proportion was found in the high category in 87 maxillary sinuses (49.15%). For the right sinus, in women, a greater presence of volume in the low category was observed in 115 sinuses (39.25%), while in men, as occurred in the left sinus, this was shown in the high category in 78 sinuses (44.07%) (Table 2). The multivariate analysis showed that the decreased right maxillary sinus volume values (<6.95) were statistically associated with female subjects (*p* = 0.004) with OR: 1.89 (95% CI: 1.23–2.91) in the right sinus when compared with males. For the left sinus, an OR: 1.87 (CI: 1.20–2.93) (*p* = 0.006) was observed (Table 3, Figure 2).

### 3.3. Nasal Septum Deviation Analysis

The presence of deviation was observed in 96.81% of the sample population, of which 60.44% showed left laterality and 39.56% right laterality. The presence of deviation was evaluated in degrees, observing a median of 11° (IQR: 7–16). Septal deviation angle was classified following Kapusuz et al.’s [1] NSD categories into mild (<9°), moderate (9–15°), and severe (≥15°) angles. In the present study, 40% of the sample had moderate angles, 33,41% mild angles, and 26.59% severe angles (Supplementary Tables S1 and S2).

### 3.4. Maxillary Sinus Volume and Degrees of Septal Deviation Analysis

**Figure 3:**
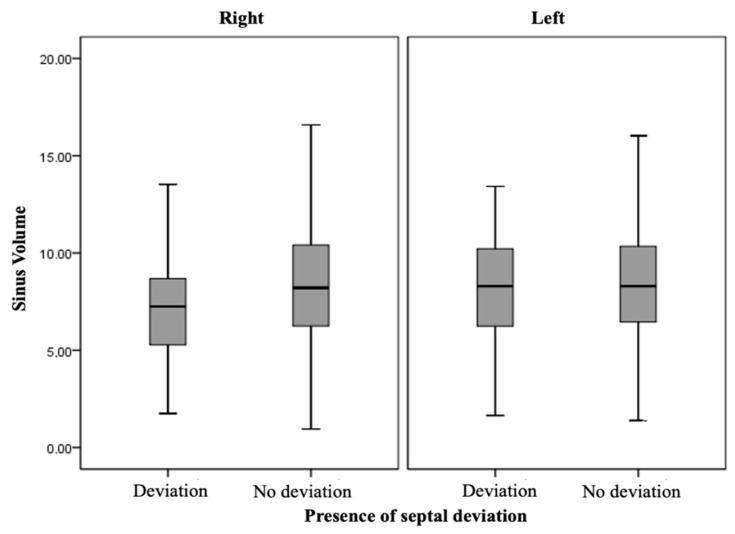
Right and left maxillary sinus volume with the presence or absence of nasal septum deviation.

**Figure 4:**
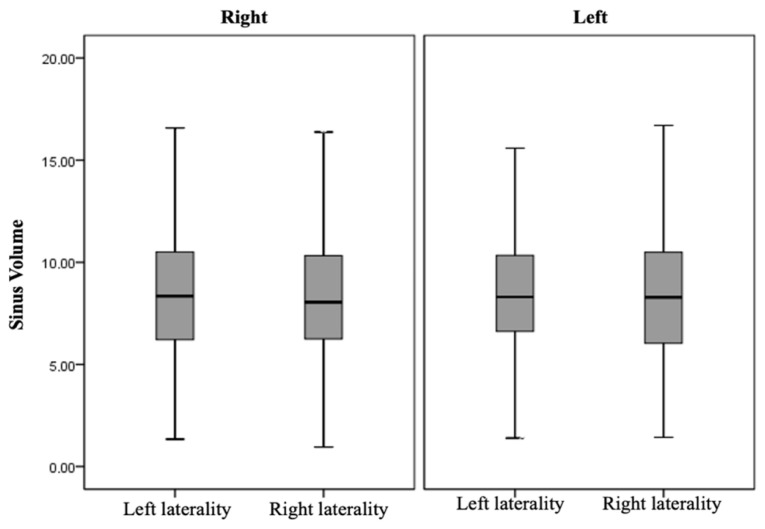
Right and left maxillary sinus volume in the presence of the nasal septum.

When comparing the volumes of the right and left maxillary sinus in the presence or absence of septal deviation with the deviation degree, no statistically significant differences were observed (Figure 3) (*p* = 0.414 for the right maxillary sinus and *p* = 0.149 for the left maxillary sinus) in the distribution with respect to volume and the presence of mild to severe angles. Thus, no positive correlation was found between the two variables compared (*p* = 0.155 and *p* = 0.197), respectively. Additionally, MSV was compared on each side according to the laterality of the deviation (right and left) (Figure 4). No statistically significant differences were observed (*p* = 0.928 for the right maxillary sinus and *p* = 0.700 for the left maxillary sinus) (Supplementary Table S2).

### 3.5. Additional Tomographic Findings

Statistically significant differences were found between both volumes and the presence of a number of right or left septa (*p* < 0.05), showing that the absence of septa could favor smaller sinus volumes. The left and right sinus volumes were statistically lower in the presence of hypoplasia (*p* < 0.05). The presence of the left turbinate was related to a smaller volume in the left sinus (*p* = 0.037). For the presence of the capsular septum, mucocele, retention cyst, hypertrophy of the inferior turbinate, thickening of Schneider’s membrane, ostium enlargement, presence of graft materials, ostium obstruction, and type of edentulism, the association was not statistically significant (*p* > 0.05). The volume results according to the different clinical characteristics are shown in Table 1. In the right and left MS, the presence of an enlarged left nasal ostium was observed (*p* = 0.89, *p* = 0.90), respectively, which could be associated with previous nasal surgery.

When evaluating the distribution of the number of MS septa according to the volume tertiles, statistically significant differences were found. For subjects with the absence of septa, MS were classified as low tertile (<6.95) (*p* < 0.05), noting that the presence of MS septa varied according to the quantity present in each sinus (0, 1, 2, 3, and 4), 1 and 2 septa were significantly more frequent in volumes greater than 9.50 both for the right sinus (*p* = 0.000 and *p* = 0.008, respectively) and the left sinus (*p* = 0.000 and *p* = 0.006), respectively. Hence, the presence of septa was associated with a decrease in the volume of MS, the presence of 1 septum on both the right (OR: 0.50, CI: 0.31–0.81) and the left side (OR: 0.60, CI: 0.36–0.98), and the presence of 1 and 2 complete right septa with both sinuses diminished (Table 3).

## 4. Discussion

The average volume of the maxillary sinus can vary to an extreme extent, between 8.6 and 24.9 cm^3^ [8,24,25]. Pneumatization of the maxillary sinus increases with edentulism [24]. Previous studies have investigated volumetric changes in the maxillary sinus in relation to dental position, NSD, and pathologies of the nasal sinuses, in addition to examining differences in the dimensions and anatomy of the maxillary sinus according to age, sex, and race. Furthermore, the width of the nasal cavity is dynamically regulated by sympathetic innervation and venous sinusoid tone, allowing for rapid adjustments through alterations in sinusoid contraction driven by functional stimulus [26]. Deviations in the convexity of the middle concha, particularly when directed laterally due to paradoxical curvature, may contribute to sinus diseases by obstructing nasal airflow, often in conjunction with septal deviation [27]. Rapid maxillary expansion may influence nasal base width and nasal valve area, impacting long-term nasal function, skeletal growth patterns, and respiratory disorders [28]. Additionally, previous studies have shown a strong correlation between maxillary sinus dimensions and midface morphology, with the maxillary sinus volume demonstrating the highest correlation with nasal width [29,30].

In the present study, no significant differences between right and left MSV have been reported, but the MSV has been shown to be significantly higher in men than in women [14,16,17,18,20]. Results of the present study showed a similar pattern, with a median and IQR of 8.18 mm^3^ (IQR: 6.2–10.33) and 8.3 mm^3^ (IQR: 6.4–10.36), respectively, and a more significant trend of increased MSV in male patients (Table 1, Figure 1). When performing a logistic regression of the present study data, on abnormal sinus volumes (<6.95) and gender, women were found to have a higher risk of presenting decreased maxillary sinuses for both the right (OR: 1.89) and the left (OR: 1.87) sides (Table 3).

Furthermore, a tendency (*p* = 0.056) was found for a decreased volume of the right maxillary sinus with partial edentulism with random multiple locations. Among other findings, a tendency towards decreased MSV (abnormal < 6.95) (*p* = 0.55) was observed for the presence of hypoplasia of the right maxillary sinus while a tendency towards decreased MSV (abnormal < 6.94), (*p* = 0.77) was observed for the presence of the right hypertrophic turbinate [7,10,11,12] (Table 1, Table 2 and Table 3).

In the present study, NSD was observed in 96.81% of the sample, with 60.44% exhibiting left laterality and 39.56% right laterality. Moderate angles were found in 40% of the sample, mild angles in 33.41%, and severe angles in 26.59%. No statistically significant differences were observed in the volumes of the right and left maxillary sinus between the presence or absence of septal deviation and the degree of deviation (*p* > 0.05). Comparing different populations, the incidence of NSD has been examined and found to range from 9% to 79.9% [6,15,17]; however, the results of these studies were influenced by the phenotypic characteristics of the population studied, which may vary according to the geographical location [5,6,14,15,17]. NSD to the right side has been reported in 36.5% of the cases and to the left side in 63.5% [12].

If we analyze the importance of this finding and the possible influence on E.N.T diseases, we find that the nasal obstruction caused by septal deviations can increase the resistance of the nasal airways and cause turbulent nasal airflow, leading to nosebleeds and recurrent sinusitis [14]. Multiple studies have supported the idea that the incidence and severity of sinusitis were correlated with the increase in the angle of NSD given a possible obstruction of the osteomeatal complex [10]. Alternatively, this could be due to the alteration of ciliary activity secondary to the modification of the airflow, thus having a clear connection between the deviation and ventilation at the level of the maxillary sinus [11]. Initially reported as a contradictory finding, in the last decade, the association was revealed after a systematic review that concluded that there was an association between NSD and the development of rhinosinusitis, although with limited impact given the multifactorial nature of the pathology [11].

Moreover, the severe septal deviation has been found in only 12.5% of certain populations [15]. The literature has shown contradictory results when analyzing the MSV and NSD relationship, and some studies showed that septal deviations had no impact on the volume of the right and left maxillary sinus [3,17,18], while others showed that ipsilateral maxillary sinus volumes in the groups with severe septum deviation were significantly smaller when compared with contralateral sinus volumes [1,3,6,14,15]. In comparison, the present study showed that NSD was present in 96.81% of the population, with 60.44% having left laterality and 39.56% having right laterality. There were no statistically significant changes in ipsilateral maxillary sinus volumes due to the presence of severe septal deviations only in 26.59% of the population with deviation angles > 15°.

In addition to the foregoing considerations, impaired nasal respiration due to septal deviations can lead to chronic mouth breathing, moderate to severe maxillary constriction, and a vertical (anterior-posterior) skeletal growth pattern [3,4]. According to the functional matrix theory, nasal airflow without alterations allows growth and development of the craniofacial structures and is a continuous stimulus for a decrease in the palate and lateral growth of the maxilla, which indicates a close relationship between nasal breathing and dentofacial morphology [3,14]. Combined septal deformity affects the septal components [20,21,22]. NSD leads to a reduction in the volume of the maxillary sinus on the deviated side; likewise, the deviation of the nasal septum with or without deviation of the hard palate causes statistically significant changes in the volume of the maxillary sinus [6]. Maxillary sinus hypoplasia may mimic sinusitis and other conditions, potentially leading to incorrect diagnoses or surgical interventions. Conversely, a laterally expanded nasal fossa extends above the maxillary alveolar bone, surpassing the typical location of teeth just beneath the antral floor. Compensatory hypertrophy, an enlargement of the inferior nasal turbinate on the concave side of NSD, aims to protect the nasal airway from cold and dry air. Compensatory hypertrophy linked to NSDs tends to persist due to bony and soft tissue thickening. Soft tissue thickening is also observed with other causes of turbinate hypertrophy [31]. Moreover, individuals with nasal septal deviation are more prone to pneumatization. Posterior pneumatization of the nasal cavity is referred to as inferior meatus pneumatization and can reach up to the second molar area, restricting the available height of the residual ridge [32]. Additionally, conditions such as nasal septum deviation, concha bullosa, paradoxical middle turbinate, deflected uncinate process, Haller cells, and maxillary hypoplasia are commonly associated with increased risk of ostium blockage or postoperative infection [32]. Therefore, maxillary sinus elevation surgery without thorough evaluation via CBCT imaging could result in inadvertent oronasal iatrogenic penetration [33,34,35].

Maxillary sinus hypoplasia (MSH) is a rare condition often mistaken for chronic sinusitis, associated with developmental issues, trauma, and potential osteomeatal complex abnormalities [34]. Awareness of MSH and its anatomical variations is crucial preoperatively due to its potential to increase infection risk and obstruct sinus mucociliary clearance. An additional result that is worth mentioning due to its relevance in sinus floor elevation surgeries is the presence of septa. Septa of the maxillary sinus is one of the most frequently evaluated findings with respect to their location, prevalence, and morphology. Maxillary sinus septa have been found in 49% of the patients, on the right (40.2%) and left (33.4%) [17], and can vary [22]. The presence of septa occurs less than 5% of the time and can completely divide the maxillary sinus into two or even five different cavities [20,22,23]. In the present study, 19 capsular septa were observed in the 940 sinuses evaluated, and these divided the maxillary sinus into up to three individual cavities. Thus, maxillary sinus septa showed statistically significant differences for the right and left sides of the maxillary sinus in the presence of one or two septa in the sample. In the logistic regression analysis, it was observed that the presence of three or more maxillary sinus septa was associated with a decreased maxillary sinus volume (abnormal < 6.95) for both the right (OR: 1.69) and the left (OR: 2.16) sides (Table 1, Table 2 and Table 3). This implies a suggestion of performing a complete assessment of the presence of septa and the volume of the maxillary sinus prior to performing surgeries that compromise the mentioned structure.

Prevalent complications during MS lift surgery include sinus membrane perforation and bleeding, while postoperative complications comprise sinus graft infections, sinus infections, and sinusitis. A comprehensive knowledge of maxillary sinus anatomy can significantly mitigate or prevent the majority of these complications [24]. Based on the findings of the present study and the evidence published by other authors [19], we suggest the use of simple manual tools such as those used here to analyze possible severe deviations of the nasal septum that may require previous ENT assessment prior to MS lift procedure as a preventive-diagnostic and therapeutic step for appropriate treatment and to exclude any possible sinus lift sinonasal complications that may lead to failure of surgery.

## 5. Conclusions

Findings in this study suggested that despite the presence of NSD in 96.81% of the population, there was no correlation with a decreased MSV. This may be associated with the low prevalence of severe nasal septum angle (>15°) (26.59%) in the population studied.

Clinicians are suggested to request a detailed CBCT analysis that includes the presence of anatomical defects and deviations of the nasal septum before performing any surgical procedure that involves the MS. Good-quality images with a large field of view (FOV) are crucial for the analysis of all anatomical structures and use of the clinical guide that establishes ENT consultations.

## Figures and Tables

**Table 1:** Comparison of left and right sinus volume according to clinical characteristics.

		Right MSV		*p* Value	Left MSV		*p* Value
		Median	IQR		Median	IQR	
Sex							
	Male	9	(6.8–11.7)	0.000	9.4	(7.2–11.4)	0.000
	Female	7.7	(5.8–9.6)		7.9	(6.1–9.7)	
Deviation							
	No	7.2	(5–9.2)	0.213	8.3	(5.5–10.4)	0.669
	Yes	8.2	(6.2–10.5)		8.3	(6.5–10.4)	
Septum laterality							
	Left	8.4	(6.2–10.6)	0.757	8.3	(6.6–10.3)	0.826
	Right	8	(6.2–10.3)		8.3	(6–10.6)	
Number of right maxillary septum							
	0	7.5 ^b^	(5.6–9.5)	0.000	7.7 ^b^	(5.8–10.1)	0.000
	1	9 ^a^	(7–10.9)		9.1 ^a^	(7.4–10.6)	
	2	10	(7.9–11.4)		10.3	(8.3–12)	
	3	9.2	(7–12.2)		8.6	(7.1–9.5)	
Number of right maxillary septum							
	0	7.7 ^b^	(5.6–9.8)	0.005	7.8 ^b^	(5.9–10)	0.000
	1	8.6 ^a^	(7.1–10.6)		9.1 ^ac^	(7.2–10.8)	
	2	9.4	(6.6–11.1)		9.6 ^b^	(7.8–11.6)	
	3	8.5	(6.5–10.6)		5.9	(4–7.8)	
	4	7.4	(3.5–11.2)		7.8	(4–11.6)	
Right Capsular							
	0	9.1	(7.2–11.1)	0.789	9.3	(7.5–10.8)	0.647
	1	10.5	(8.6–11.2)		8.8	(7.9–10.7)	
	2	9.3	(9.3–9.3)		11	(11–11)	
Left Capsular							
	0	8.8	(7–10.9)	0.118	9.2	(7.4–11.1)	0.285
	1	7.4	(6.8–8.2)		8.5	(7.1–10.2)	
	2	6.1	(5.7–6.4)		6.2	(4.5–7.8)	
	3	10.6	(10.6–10.6)		7.8	(7.8–7.8)	
Right turbinate							
	Absence	8.2	(6.3–10.5)	0.202	8.4	(6.5–10.5)	0.037
	Presence	7.3	(4.8–9.5)		7.3	(4.2–9.3)	
Left turbinate							
	Absence	8.2	(6.3–10.3)	0.350	8.3	(6.5–10.4)	0.545
	Presence	7.1	(5–10.6)		7.8	(5.5–10.7)	
Right Ostium							
	Absence	8.2	(6.2–10.3)	0.891	8.3	(6.5–10.4)	0.918
	Presence	7.7	(6.7–9.5)		9.1	(7.3–9.7)	
Left Ostium							
	Absence	8.2	(6.2–10.5)	0.576	8.3	(6.5–10.4)	0.389
	Presence	8.2	(5.5–9.3)		8.2	(6.6–9)	
Right Hypoplasia							
	Absence	8.2	(6.2–10.4)	0.016	8.3	(6.5–10.4)	0.017
	Presence	1.5	(1–2.1)		2	(1.7–2.2)	
Left Hypoplasia							
	Absence	8.2	(6.2–10.3)	0.502	8.3	(6.5–10.4)	0.003
	Presence	7.2	(2.1–10.7)		1.4	(0.5–2.2)	
Location of Edentulism							
	Right	8.2	(6.3–10.7)	0.056	8.8	(6–10.7)	0.417
	Left	7.4	(5.8–8.9)		8.1	(6.1–9.5)	
	Bilateral	7.7	(5.8–10.6)		8.2	(6.4–10.5)	
	Random multiple	8.7	(6.8–11.2)		8.5	(6.3–11.1)	

The statistical significance of variables (^a^, ^b^, ^c^) was analyzed using Kruskal-Wallis test and Mann-Whitney U test. ^a^ indicates statistically significant difference in the volume of the maxillary sinus in the presence of the first category of analysis (abscense of maxillary septum), ^b^ indicates significant difference in the volume of the maxillary sinus in the presence of the Second category of analysis (presence of 1 maxillary septum), and so on.

**Table 2:** Distribution of clinical characteristics according to high volume, medium-low volume of the right and left sinuses.

		Right Sinus Volume						*p* Value	Left Sinus Volume						*p* Value
		Low (<6.95)		Medium (6.96–9.50)		High (<9.50)			Low (<6.95)		Medium (6.96–9.50)		High (<9.50)		
		*n*	(%)	*n*	(%)	*n*	(%)		*n*	(%)	*n*	(%)	*n*	(%)	
Sex															
	Male	51	(28.81)	48	(27.12)	78	(44.07)	0.000	43	(24.29)	47	(26.55)	87	(49.15)	0.000
	Female	115	(39.25)	101	(34.47)	77	(26.28)		101	(34.47)	115	(39.25)	77	(26.28)	
Deviation															
	No	6	(40.0)	6	(40.0)	3	(20.0)	0.542	4	(26.67)	5	(33.33)	6	(40.00)	0.904
	Yes	160	(35.16)	143	(31.43)	152	(33.41)		140	(30.77)	157	(34.51)	158	(34.73)	
Septum laterality															
	Left	95	(34.55)	88	(32.00)	92	(33.45)	0.928	82	(29.82)	99	(36.00)	94	(34.18)	0.700
	Right	65	(36.11)	55	(30.56)	60	(33.33)		58	(32.22)	58	(32.22)	64	(35.56)	
Number of right maxillary septum															
	0	123	(44.40)	86	(31.05)	68	(24.55)	0.000	111	(40.07)	89	(32.13)	77	(27.80)	0.000
	1	37	24.34)	50	(32.89)	65	(42.76)		30	(19.74)	58	(38.16)	64	(42.11)	
	2	4	11.76)	11	(32.35)	19	(55.88)		2	(5.88)	11	(32.35)	21	(61.76)	
	3	2	28.57)	2	(28.57)	3	(42.86)		1	(14.29)	4	(57.14)	2	(28.57)	
Number of left maxillary septum															
	0	116	(42.65)	79	(29.04)	77	(28.31)	0.008	101	(37.13)	94	(34.56)	77	(28.31)	0.006
	1	37	(24.34)	59	(38.82)	56	(36.84)		34	(22.37)	54	(35.53)	64	(42.11)	
	2	11	(26.19)	11	(26.19)	20	(47.62)		7	(16.67)	13	(30.95)	22	(52.38)	
	3	1	(50.00)	0	(0.00)	1	(50.00)		1	(50.00)	1	(50.00)	0	(0.00)	
	4	1	(50.00)	0	(0.00)	1	(50.00)		1	(50.00)	0	(0.00)	1	(50.00)	
Left Capsular															
	0	45	(24.46)	63	(34.24)	76	(41.30)	0.046	40	(21.74)	61	(33.15)	83	(45.11)	0.622
	1	3	(27.27)	7	(63.64)	1	(9.09)		2	(18.18)	5	(45.45)	4	(36.36)	
	2	2	(100.00)	0	(0.00)	0	(0.00)		1	(50.00)	1	(50.00)	0	(0.00)	
	3	0	(0.00)	0	(0.00)	1	(100.00)		0	(0.00)	1	(100.00)	0	(0.00)	
Left Ostium															
	Absence	163	(35.28)	146	(31.60)	153	(33.12)	0.881	141	(30.52)	157	(33.98)	164	(35.50)	0.090
	Presence	3	(37.50)	3	(37.50)	2	(25.00)		3	(37.50)	5	(62.50)	0	(0.00)	
Right Hypoplasia															
	Absence	164	(35.04)	149	(31.84)	155	(33.12)	0.159	142	(30.34)	162	(34.62)	164	(35.04)	0.103
	Presence	2	(100.00)	0	(0.00)	0	(0.00)		2	(100.00)	0	(0.00)	0	(0.00)	
Left Hypoplasia															
	Absence	165	(35.33)	148	(31.69)	154	(32.98)	0.997	141	(30.19)	162	(34.69)	164	(35.12)	0.033
	Presence	1	(33.33)	1	(33.33)	1	(33.33)		3	(100.00)	0	(0.00)	0	(0.00)	
Edentulism location															
	Right	21	(33.33)	17	(26.98)	25	(39.68)	0.068	21	(33.33)	18	(28.57)	24	(38.10)	0.412
	Left	29	(42.65)	26	(38.24)	13	(19.12)		23	(33.82)	28	(41.18)	17	(25.00)	
	Bilateral	21	(42.00)	12	(24.00)	17	(34.00)		18	(36.00)	13	(26.00)	19	(38.00)	
	Random Multiple	17	(26.15)	21	(32.31)	27	(41.54)		17	(26.15)	23	(35.38)	25	(38.46)	

The statistical analysis was conducted using both the Chi-squared test and Fisher’s exact test.

**Table 3:** Logistic regression presence of decreased volume adjusted to associated clinical characteristics.

		Decreased Right Maxillary Sinus			Decreased Left Maxillary Sinus		
		OR	IC 95%	*p* Value	OR	IC 95%	*p* Value
Gender							
	*Male*		1			1	
	*Female*	1.89	(1.23–2.91)	0.004	1.87	(1.20–2.93)	0.006
Numbers of right septa							
	*0*		1			1	
	*1*	0.46	(0.28–0.73)	0.001	0.39	(0.23–0.64)	0.000
	*2*	0.19	(0.06–0.60)	0.005	0.11	(0.02–0.49)	0.004
	*3*	0.64	(0.11–3.60)	0.614	0.31	(0.03–2.82)	0.304
Numbers of left septa							
	*0*		1			1	
	*1*	0.50	(0.31–0.81)	0.005	0.60	(0.36–0.98)	0.044
	*2*	0.79	(0.35–1.79)	0.578	0.60	(0.24–1.53)	0.295
	*3*	1.69	(0.08 -32.0)	0.726	2.16	(0.10–43.6)	0.613
	*4*	1.23	(0.71- 21.1)	0.885	1.58	(0.08–27.8)	0.753
Left obstruction							
	*Absence*		1			1	
	*Presence*	0.25	(0.05–1.17)	0.080	0.30	(0.06–1.45)	0.137
Right turbinate							
	*Absence*		1			1	
	*Presence*	0.25	(0.05–1.17)	0.080	0.91	(0.43–1.94)	0.825

## Data Availability

The data presented in this study are available on request from the corresponding author. The data are not publicly available due to ethical restrictions.

## Supplementary Materials

The following supporting information can be downloaded at: https://www.mdpi.com/article/10.3390/diagnostics14060647/s1, Table S1: Descriptive analysis of the classification of the septal deviation angulations; Table S2: Bivariate analysis between the volume of the right and left maxillary sinus with the degrees of septal deviation.

## References

[1] Kapusuz Gencer Z., Özkırış M., Okur A., Karaçavuş S., Saydam L. (2013). The effect of nasal septal deviation on maxillary sinus volumes and development of maxillary sinusitis. Eur. Arch. Oto-Rhino-Laryngol..

[2] Kawarai Y., Fukushima K., Ogawa T., Nishizaki K., Gunduz M., Fujimoto M., Masuda Y. (1999). Volume quantification of healthy paranasal cavity by three-dimensional CT imaging. Acta Oto-Laryngol..

[3] Kucybała I., Janik K.A., Ciuk S., Storman D., Urbanik A. (2017). Nasal septal deviation and concha bullosa–do they have an impact on maxillary sinus volumes and prevalence of maxillary sinusitis?. Pol. J. Radiol..

[4] Lawson W., Patel Z.M., Lin F.Y. (2008). The development and pathologic processes that influence maxillary sinus pneumatization. Anat. Rec. Adv. Integr. Anat. Evol. Biol. Adv. Integr. Anat. Evol. Biol..

[5] Przystańska A., Kulczyk T., Rewekant A., Sroka A., Jończyk-Potoczna K., Lorkiewicz-Muszyńska D., Gawriołek K., Czajka-Jakubowska A. (2018). Introducing a simple method of maxillary sinus volume assessment based on linear dimensions. Ann. Anat. Anat. Anz..

[6] Sapmaz E., Kavaklı A., Sapmaz H.I., Ögetürk M. (2018). Impact of hard palate angulation caused by septal deviation on maxillary sinus volume. Turk. Arch. Otorhinolaryngol..

[7] Wolf G., Anderhuber W., Kuhn F. (1993). Development of the paranasal sinuses in children: Implications for paranasal sinus surgery. Ann. Otol. Rhinol. Laryngol..

[8] Oz A.Z., Oz A.A., El H., Palomo J.M. (2017). Maxillary sinus volume in patients with impacted canines. Angle Orthod..

[9] Tassoker M., Magat G., Lale B., Gulec M., Ozcan S., Orhan K. (2020). Is the maxillary sinus volume affected by concha bullosa, nasal septal deviation, and impacted teeth? A CBCT study. Eur. Arch. Oto-Rhino-Laryngol..

[10] Bingham B., Wang R.G., Hawke M., Kwok P. (1991). The embryonic development of the lateral nasal wall from 8 to 24 weeks. Laryngoscope.

[11] Collet S., Bertrand B., Cornu S., Eloy P., Rombaux P. (2001). Is septal deviation a risk factor for chronic sinusitis? Review of literature. Acta Oto-Rhino-Laryngol. Belg..

[12] Orlandi R.R. (2010). A systematic analysis of septal deviation associated with rhinosinusitis. Laryngoscope.

[13] Al-Rawi N.H., Uthman A.T., Abdulhameed E., Al Nuaimi A.S., Seraj Z. (2019). Concha bullosa, nasal septal deviation, and their impacts on maxillary sinus volume among Emirati people: A cone-beam computed tomography study. Imaging Sci. Dent..

[14] Akay G., Yaman D., Karadağ Ö., Güngör K. (2020). Evaluation of the Relationship of Dimensions of Maxillary Sinus Drainage System with Anatomical Variations and Sinusopathy: Cone-Beam Computed Tomography Findings. Med. Princ. Pr..

[15] Aktuna Belgin C., Colak M., Adiguzel O., Akkus Z., Orhan K. (2019). Three-dimensional evaluation of maxillary sinus volume in different age and sex groups using CBCT. Eur. Arch. Otorhinolaryngol..

[16] Kalabalık F., Tarım Ertaş E. (2019). Investigation of maxillary sinus volume relationships with nasal septal deviation, concha bullosa, and impacted or missing teeth using cone-beam computed tomography. Oral Radiol..

[17] Mladina R., Cujić E., Subarić M., Vuković K. (2008). Nasal septal deformities in ear, nose, and throat patients: An international study. Am. J. Otolaryngol..

[18] Orhan I., Ormeci T., Aydin S., Altin G., Urger E., Soylu E., Yilmaz F. (2014). Morphometric analysis of the maxillary sinus in patients with nasal septum deviation. Eur. Arch. Oto-Rhino-Laryngol..

[19] Biafora M., Bertazzoni G., Trimarchi M. (2014). Maxillary sinusitis caused by dental implants extending into the maxillary sinus and the nasal cavities. J. Prosthodont..

[20] Kim S.J., Park J.S., Kim H.T., Lee C.H., Park Y.H., Bae J.H. (2016). Clinical features and treatment outcomes of dental implant-related paranasal sinusitis: A 2-year prospective observational study. Clin. Oral Implant. Res..

[21] Testori T., Weinstein T., Taschieri S., Wallace S.S. (2019). Risk factors in lateral window sinus elevation surgery. Periodontology 2000.

[22] Van den Bergh J.P., ten Bruggenkate C.M., Disch F.J., Tuinzing D.B. (2000). Anatomical aspects of sinus floor elevations. Clin. Oral Implant. Res..

[23] Gies M., Kalender W.A., Wolf H., Suess C., Madsen M.T. (1999). Dose reduction in CT by anatomically adapted tube current modulation. I. Simulation studies. Med. Phys..

[24] Möhlhenrich S.C., Heussen N., Peters F., Steiner T., Hölzle F., Modabber A. (2015). Is the maxillary sinus really suitable in sex determination? A three-dimensional analysis of maxillary sinus volume and surface depending on sex and dentition. J. Craniofacial Surg..

[25] Ulm C., Solar P., Gselimann B., Matejka M., Watzek G. (1995). The edentulous maxillary alveolar process in the region of the maxillary sinus—A study of physical dimension. Int. J. Oral Maxillofac. Surg..

[26] Mygind N., Dahl R. (1998). Anatomy, physiology and function of the nasal cavities in health and disease. Adv. Drug Deliv. Rev..

[27] Papadopoulou A.-M., Chrysikos D., Samolis A., Tsakotos G., Troupis T. (2021). Anatomical variations of the nasal cavities and paranasal sinuses: A systematic review. Cureus.

[28] Neeley W.W., Edgin W.A., Gonzales D.A. (2007). A review of the effects of expansion of the nasal base on nasal airflow and resistance. J. Oral Maxillofac. Surg..

[29] Alhazmi A. (2020). Association between maxillary sinus dimensions and midface width: 2-D and 3-D volumetric cone-beam computed tomography cross-sectional study. J. Contemp. Dent. Pr..

[30] Song S.Y., Hong J.W., Roh T.S., Kim Y.O., Kim D.W., Park B.Y. (2009). Volume and distances of the maxillary sinus in craniofacial deformities with midfacial hypoplasia. Otolaryngol. -Head. Neck Surg..

[31] Shetty S.R., Al Bayatti S.W., Al-Rawi N.H., Marei H., Reddy S., Abdelmagyd H.A., Narasimhan S., Al Kawas S., Mathew A. (2021). Analysis of inferior nasal turbinate width and concha bullosa in subjects with nasal septum deviation: A cone beam tomography study. BMC Oral Health.

[32] Park W.B., Kim Y.J., Kang K.L., Lim H.C., Han J.Y. (2020). Long-term outcomes of the implants accidentally protruding into nasal cavity extended to posterior maxilla due to inferior meatus pneumatization. Clin. Implant. Dent. Relat. Res..

[33] Ilie A.C., Jianu A.M., Rusu M.C., Mureșan A.N. (2022). Anatomical Changes in a Case with Asymmetrical Bilateral Maxillary Sinus Hypoplasia. Medicina.

[34] Sirikci A., Bayazit Y., G÷m÷sburun E., Bayram M., Kanlikana M. (2001). A new approach to the classification of maxillary sinus hypoplasia with relevant clinical implications. Surg. Radiol. Anat..

[35] Güngör G., Okur N., Okur E. (2016). Uncinate Process Variations and Their Relationship with Ostiomeatal Complex: A Pictorial Essay of Multidedector Computed Tomography (MDCT) Findings. Pol. J. Radiol..

