# Supplementary material for: Relationship of Maxillary Sinus Volume and Nasal Septum Deviation: A Cone Beam Computed Tomography Study
Source: Diagnostics (Basel). 2024 Mar 19;14(6):647. doi: 10.3390/diagnostics14060647 (PMC10969206; doi:10.3390/diagnostics14060647)
Supplement: Supplementary file 1 [file diagnostics-14-00647-s001.zip › diagnostics-2876927-supplementary.pdf]

Supplemental data

Tables S1. Descriptive analysis of the classification of the septal deviation angulations

		n	%
Septal Deviation	<9	152	33.41%
Angulation	9-15	182	40.00%
Classification	>15	121	26.59%

Table S2. Bivariate analysis between the volume of the right and left maxillary sinus with the degrees of septal deviation

	Degrees of Septal Deviation			
	<9<br>Mean<br>DS	9-15<br>Mean<br>DS	>15<br>Mean<br>DS	p Value
Right Maxillary Sinus	8.50 ± 2.95	8.10 ± 3.09	8.48 ± 3.09	0.414
Left Maxillary Sinus	8.85 ± 3.05	8.18 ± 3.01	8.59 ± 3.26	0.149
